# Exposure to Cry1 Toxins Increases Long Flight Tendency in Susceptible but Not in Cry1F-Resistant Female *Spodoptera frugiperda* (Lepidoptera: Noctuidae)

**DOI:** 10.3390/insects15010007

**Published:** 2023-12-22

**Authors:** Caroline P. De Bortoli, Rafael F. Santos, Giordano J. Assirati, Xiaocun Sun, Lucas Hietala, Juan Luis Jurat-Fuentes

**Affiliations:** 1 Department of Entomology and Plant Pathology, University of Tennessee, Knoxville, TN 37996, USA; cplacidi@utk.edu (C.P.D.B.); rferrei1@utk.edu (R.F.S.); gassirati@hotmail.com (G.J.A.); lhietal1@utk.edu (L.H.); 2 Research Computing Support, Office of Information Technology, University of Tennessee, Knoxville, TN 37996, USA; xsun@utk.edu

**Keywords:** fall armyworm, *Spodoptera frugiperda*, migratory behavior, induced flight, dispersion, flight mill, resistance, Cry1Ac, Cry1F

## Abstract

**Simple Summary:**

The fall armyworm (*Spodoptera frugiperda*) is a devastating pest for multiple crops, especially corn. Transgenic corn producing Cry and Vip3A insecticidal proteins from the bacterium *Bacillus thuringiensis* (Bt corn) controls *S. frugiperda*, although cases of practical resistance to Cry proteins have been reported in the Caribbean, and North and South America. The long-distance migratory flight capacity of *S. frugiperda* is of concern given its ongoing global spread and the possibility of resistance dispersal. In this study, we used rotational flight mills to test the effect of exposure to Cry1Ac and Cry1F proteins on flight tendency in *S. frugiperda* moths, including susceptible and Cry1F-resistant populations. Results support that generally lethal exposure of *S. frugiperda* larvae to Cry proteins in susceptible insects increases tendency for longer flights in female moths, while this behavior was not observed in Cry1F-resistant moths. This information helps understand factors affecting the migratory spread of *S. frugiperda* and its implications for resistance management of Bt crops.

**Abstract:**

The fall armyworm (JE Smith) (*Spodoptera frugiperda*) is a polyphagous pest targeted by selected Cry and Vip3A insecticidal proteins from the bacterium *Bacillus thuringiensis* (Bt) that are produced in transgenic Bt corn and cotton. Available evidence suggests that sublethal larval exposure to Cry1Ac increases flight activity in adult *Spodoptera* spp. However, it is not known whether this effect is also observed in survivors from generally lethal exposure to Cry1Ac. Moreover, while multiple cases of field-evolved resistance to Bt proteins have been described in the native range of *S. frugiperda*, the effect of resistance on flight behavior has not been examined. Long-distance migratory flight capacity of *S. frugiperda* is of concern given its ongoing global spread and the possibility that migrants may be carrying resistance alleles against pesticides and Bt crops. In this study, we used rotational flight mills to test the effects of generally lethal exposure to Cry1Ac in susceptible and sublethal exposure in Cry1F-resistant *S. frugiperda* strains. The results detected altered pupal weight after larval feeding on diet containing Cry proteins, which only translated in significantly increased tendency for longer flights in female moths from the susceptible strain. This information has relevant implications when considering current models and assumptions for resistance management of Bt crops.

## 1. Introduction

The fall armyworm (J.E. Smith), *Spodoptera frugiperda*, is a highly polyphagous pest [1], causing economically relevant damage to corn, cotton, and sorghum [2]. Since 2016, this insect has emerged from being a pest of incidence in its native range in the tropical Americas, to a pest threatening food and fiber production globally (see updated distribution at https://gd.eppo.int/taxon/LAPHFR/distribution, accessed on 15 November 2023). Originally detected as an invasive pest in West and Central Africa [3], it was quickly confirmed in most of sub-Saharan Africa [4], India [5], Southeast Asia [6,7,8], Oceania [9], and more recently in Europe [10,11]. In the USA, *S. frugiperda* follows an annual northward migration over several generations from diapausing populations in the warmer southern regions of Florida and Texas [12]. A similar pattern of migration from diapausing “hot spots” offering optimal climate and host availability for *S. frugiperda* persistence has been proposed in its invasive range [13,14]. Increased population levels and continued migration from these “hot spots”, together with increased suitable geographic locations globally due to climate change [15], highlight the increasing risk of continued expansion of *S. frugiperda*. Moreover, *S. frugiperda* is notorious for its ability to evolve resistance to pesticides, with 250 cases of documented resistance to multiple insecticide groups [16]. Importantly, spread of this resistance through migratory routes would further exacerbate the economic impacts from continued *S. frugiperda* expansion.

Transgenic corn producing the Cry1F insecticidal protein from the bacterium *Bacillus thuringiensis* was commercialized as an effective tool in controlling *S. frugiperda* in its native range [17,18]. However, populations of *S. frugiperda* in Puerto Rico [19], Florida and North Carolina [20], Brazil [21], and Argentina [22] have evolved practical resistance to Cry1F corn. When studied, the mechanism of practical resistance to Cry1F corn involves alterations in an ABC transporter gene (*SfABCC2*) that disrupt the functional Cry1F receptor role of this protein in the midgut of *S. frugiperda* larvae [23,24,25,26]. Remarkably, genetic alterations in *SfABCC2* do not appear to result in relevant fitness costs in the laboratory [27,28]. This lack of fitness costs may enable the maintenance of standing genetic variation in *S. frugiperda* populations, contributing to the fast development and continued high frequency of resistance observed in Puerto Rico, even after voluntary withdrawal of Cry1F corn from the local market [23,29]. Contrary to the expected migration of *S. frugiperda* island-hoping through the Caribbean into Florida [12], the allele linked to Cry1F resistance in Puerto Rico is not found elsewhere [24,26,30]. However, the potential effects of resistance to Cry1F on *S. frugiperda* flight tendency have not been addressed to date. 

Insect flight dispersal is the result of interactions between dispersal ability (whether the insect has morphological attributes for flight) and dispersal propensity (whether the insect decides to disperse or remain in the natal environment) [31]. Suboptimal (poor quality nutrition) or sublethal (low toxicity) larval food may induce insect dispersal when fitness costs are lower than maintaining future offspring on a low-quality host [32]. Thus, a previous report presented data supporting increased flight distance by *Spodoptera exigua* (Hübner) (Lepidoptera: Noctuidae) moths when they were exposed to sublethal concentrations of Cry1Ac as larvae [33]. However, whether this behavior is maintained in insects surviving a generally lethal exposure (i.e., resulting in 85% mortality) to Cry proteins or in insects with practical resistance to Bt crops is not known. Feeding on Bt crops would represent a sublethal exposure to Cry proteins for resistant insects. Potential increased flight tendency in Cry-resistant insects after this sublethal exposure could have important consequences for resistance management since the current high dose/refuge IRM strategy for Bt crops assumes random mating between susceptible individuals from refugia and nearby resistant individuals emerging from Bt crops. 

In this study, we tested for potential changes in flight tendency in *S. frugiperda* moths that as larvae were exposed to diet containing generally lethal levels of Cry1Ac compared to a control diet, and extended this analysis to sublethal Cry1Ac and Cry1F treatments in Cry1F-resistant *S. frugiperda*. Effects of exposure on pupal weight, as a proxy for developmental effects, total flown distance, and resident versus migratory behavior, were measured in newly eclosed moths using rotational flight mills and compared considering strain resistance phenotype and moth sex.

## 2. Materials and Methods

### 2.1. Insects

The strains of *S. frugiperda* used in this study were maintained in the Insect Molecular Pathology and Resistance (IMPaR) laboratory at the University of Tennessee (Knoxville) under rearing conditions of 25 ± 1 °C, a photoperiod of 16 h light and 8 h dark, and relative humidity 70 ± 5%. Eggs of a Bt-susceptible strain of *S. frugiperda* were purchased from Benzon Inc. (Carlislse, PA, USA). The Cry1F-resistant strain used in this study (456LS4D) was derived after multiple crossings of *S. frugiperda* strain 456, originated from collections in Puerto Rico and displaying resistance to Cry1F [34], to the Benzon strain. Offspring from these crosses were reared and sib-mated to generate an F2 progeny that was selected with a discriminatory dose of Cry1F killing heterozygotes for resistance [35]. These crossing–selection cycles were performed for a total of 4 times in generating a strain (456LS4D) that is more closely related genetically to the Benzon strain than the parental 456 strain but preserving resistance alleles. Larvae from both strains were reared on meridic diet (Frontier Scientific Services, beet armyworm diet without antibiotic), and adults were maintained on 10% sucrose delivered in a wet cotton ball. 

### 2.2. Cry Proteins

Insecticidal proteins were produced in *B. thuringiensis* strain HD73 harboring the *cry1Ac* gene, obtained from the *Bacillus* Genetic Stock Center (Columbus, OH, USA) and a previously described recombinant B. thuringiensis strain harboring the *cry1F* gene [36]. Bacterial cultures confirmed to be at the sporulation stage by microscopic examination were centrifuged (14,981× *g*, 4 °C for 10 min) and pellets containing spores and crystals were washed thrice with 1 M NaCl plus 0.1% Triton X-100 and twice with distilled water. Crystals in the final pellet containing Cry proteins were solubilized in 50 mM Na_2_CO_3_, 0.1% β-mercaptoethanol, 0.1 M NaCl (pH 10.5) overnight at 30 °C. After centrifugation (27,167× *g* for 30 min), the solubilized Cry protoxins in the supernatant were activated with bovine trypsin and then purified using anion exchange chromatography as described elsewhere [37]. Purity of activated Cry1Ac and Cry1F in eluted fractions was examined using SDS-10% PAGE before quantification with Qubit fluorometry (Invitrogen, Waltham, MA, USA) and stored at −80 °C until use (<2 months).

### 2.3. Larval Exposure to Cry Proteins and Pupal Weight

Exposure to Cry1Ac and Cry1F proteins was on individual wells of bioassay trays (128-well bioassay trays, C-D International, Pitman, NJ, USA) containing meridic diet. After the diet was dispensed in individual wells and solidified, it was covered with a solution containing purified Cry toxins, as described elsewhere [36]. Meridic diet covered with dilution solution (water) was used as a control diet. A sublethal concentration of Cry1Ac and Cry1F (4.75 µg/cm^2^), defined as not inducing increased mortality when compared to the control diet under rearing conditions, was estimated for the 456LS4D strain from preliminary and reported bioassays [38,39]. This concentration of Cry1Ac was more than double the LC_50_ for Cry1Ac in Benzon (2 µg/cm^2^), and induced 85% mortality in Benzon larvae. Based on these observations, we refer the treatment with this dose of Cry1Ac against Benzon larvae as “generally lethal”. Newly hatched *S. frugiperda* larvae from the Benzon and 456LS4D strains were individually exposed to the treated artificial diet for 7 days, and then survivors were transferred to a new diet cup containing fresh meridic diet without Cry toxin and kept there until reaching pupal stage. Pupae were weighed, their sex determined from external discriminatory features [40], and then transferred to a new empty cup and incubated under rearing conditions until adult emergence. 

### 2.4. Rotary Flight Mill (RFM) Experiments

Rotary flight mills (RFMs) were developed based on previous models [41,42]. Briefly, RFMs use a magnetic levitation mechanism between Teflon bearings that reduces friction as the flying insect spins around the mill [43]. A unipolar magnetic Hall-effect magnetic sensor at the top of the main RFM Teflon rod detects the change in magnetic polarity from passage of a small magnet attached to the base of the rotating bearing when the mill is in motion.

Recently emerged *S. frugiperda* adults (12–18 h post-emergence) were inspected for any physical deformation preventing flight and kept at room temperature until flight test performance. A total of 20 moths of the same sex, each in an individual flight mill, were tested simultaneously. Individual *S. frugiperda* moths were placed for 10–15 s in a 1 oz plastic cup connected at the bottom to the opening of a 50 mL falcon tube containing dry ice as source of CO_2_ gas anesthetic. Anesthetized moths were cleaned of dorsal scales on their thoracic segment and a double-face Velcro placed in the cleaned area, while the other Velcro face was attached to a hard point. The hard point was then placed at one side of the RFM flight arm using an entomological pin. The cap of a microfuge tube was used as counterweight on the other side of the flight arm. Temperature conditions used during flight recordings ranged between 19 and 26 °C and 50 and 70% humidity. This range of temperature conditions were previously reported as optimal for *S. frugiperda* tethered flight in RFMs [44,45]. A total of 20 RFMs were operated simultaneously, continuously recording moth flight activity for 24 h. The publicly available custom MothMill v1.0 and MillCollector v1.0 software programs (GitHub: https://github.com/nchietala/rpi-sensor-controller, accessed on 15 November 2023) were used in collecting and integrating data from multiple RFM mills, obtaining average flight parameters for each *S. frugiperda* strain and treatment [46].

### 2.5. Data Analysis

All raw data analyzed in this work is presented in Table S1. Calculation of statistical means and corresponding standard errors, and testing for significant differences in weight and total flown distance was carried out using the SigmaPlot v11.0 Build 11.2.0.5 (Systat Software, San Jose, CA, USA) software. Normality of data was tested using the Shapiro–Wilk test (passed if *p* > 0.05), and differences between strain, sex and treatment were tested using the Mann–Whitney Rank Sum test (*p* < 0.05) when the normality tests failed. The mean pupal weight for each *S. frugiperda* strain was estimated from 68 (Benzon) and 96 (456LS4D) individuals. Pupal weight data for comparison of sex in 456LS4D (*p* = 0.256) and for treatment in Benzon males (*p* = 0.071) that passed the normality tests and differences were tested using a *t*-test (*p* < 0.05). Differences in the effect of diet (control, Cry1Ac and Cry1F) on weight and flight behavior in the 456LS4D strain were estimated using a one-way ANOVA with rank data transformation, as diagnostic analysis exhibited violation of normality assumptions using the Shapiro–Wilk test. In this case, a post hoc multiple (pairwise) comparison was performed with Dunn’s adjustments (*p* < 0.05) to test for the existence of differences and their significance was tested using a *t*-test (*p* < 0.05). In the case of female pupal weights in 456LS4D, the data for treatment passed the normality test and were analyzed using a one-way ANOVA with post hoc pairwise comparisons with a Holm–Sidak test (*p* < 0.05). Binary fly tendency data (resident versus migratory) were analyzed for the impact of strain, sex and diet, respectively, using a logistic regression with weight as the controlling covariate using SAS 9.4 TS1M8 for windows ×64 (SAS institute Inc., Cary, NC, USA) and statistical significance identified at *p* < 0.05.

## 3. Results and Discussion

### 3.1. Effects of Sublethal Treatment on Pupal Weight

Weights of pupae when larvae from the Benzon and 456LSD strains where reared on control or Cry toxin diet are shown in Table 1. Comparisons of pupal weight when larvae were fed the control diet showed no effects of sex in the Benzon (*p* = 0.3654) and 456LS4D (*p* = 0.2717) strains. In contrast, pupal weight differences between strains were significant (*p* < 0.0001), with heavier pupae in the 456LS4D (186.8 ± 2.8 mg) compared to the Benzon (173.1 ± 2.7 mg) strain. This observation was unexpected considering that the 456LS4D strain had been crossed to Benzon and then re-selected for resistance to Cry1F to develop a more closely related genetically line to Benzon that preserved resistance alleles. The observed discrepancy in growth between the strains on the control diet suggests that genetic factors controlling developmental phenotypes may have co-segregated with the Cry1F resistance phenotype in 456LS4D or reflect remaining genetic dissimilarity between the strains. In fact, while differences in pupal weight were not previously detected between the parental strain to 456LS4D (456) and Benzon when grown on the control diet, increased pupal weight was observed in progeny from 456 and Benzon crosses [27]. Thus, the continued backcrossing of 456 to Benzon and subsequent reselection process used to generate the 456LS4D strain could have co-selected alleles affecting development. Similarly, co-selection of increased vigor and resistance to gene silencing by RNA interference were proposed to explain marginally reduced susceptibility to Bt toxins [47,48]. 

Benzon female larvae surviving exposure to a generally lethal level of Cry1Ac resulted in heavier pupae when compared to pupae from larvae reared on the control diet (*p* = 0.0067). In contrast, no significant impact of exposure to Cry1Ac was detected on the weight of male Benzon pupae (*p* = 0.3918). Increased pupal weight after exposure to Cry proteins is uncommon in the literature, where exposure typically results in reduced pupal weight [33,49,50,51]. However, it was previously reported in *Plutella xylostella* (L.) (Lepidoptera: Plutellidae) resistant to Cry1Ac [52]. One potential explanation offered to explain this higher pupal weight was the increased food consumption and potential nutritional benefits observed in the resistant larvae from feeding on a diet containing an inactive Cry protein [52]. This was shown not to be the case in other insect strains resistant to Bt proteins [53]. It is important to consider that the generally lethal amounts of Cry1Ac used in our study could approximate exposure to the high levels of Cry proteins produced by Bt crops. In this regard, *Helicoverpa zea* (Boddie) (Lepidoptera: Noctuidae) pupae collected from Bt corn plots were lighter than those collected from a non-Bt corn refuge [54]. However, field populations of *H. zea* have evolved resistance to some of the proteins in the tested Bt corn hybrids in that study [55], which could have affected the physiological response to exposure.

In contrast to the increased female pupal weight observed in Benzon, sublethal exposure to Cry1Ac in 456LS4D resulted in significantly lower pupal weight in both females (*p* = 0.0002) and males (*p* = 0.0002), when compared to larvae fed the control diet. These observations suggest incomplete resistance [56] and mirror reports of reduced pupal weight after sublethal exposure to Cry proteins in the literature, including *S. exigua* exposure to Cry1Ac [33,49,50,51]. The discrepancy in effects between Benzon and 456LS4D strains probably relates to different pleiotropic effects exerted by the concentration of Cry1Ac toxin used in this study, which represents a generally lethal and sublethal treatment for Benzon and 456LS4D, respectively. In addition, the Cry1F resistance mechanism in 456LS4D and remaining genetic dissimilarity could have also affected the developmental response after exposure to Cry1Ac and explain divergence with the Benzon strain. 

The Cry1F protein is highly active against *S. frugiperda* [18]. The Cry1F concentration used in this study was a sublethal treatment for 456LS4D but caused 100% mortality in Benzon larvae. In contrast to the reduced pupal weight after Cry1Ac exposure, rearing 456LS4D larvae on diet containing Cry1F did not result in differences in female (*p* = 0.1723) or male (*p* = 0.2586) pupal weight compared to the control diet. This discrepancy could be explained by distinct effects induced by Cry1Ac and Cry1F on 456LS4D larvae. Weight was significantly reduced in both female (*p* < 0.0001) and male (*p* < 0.0001) pupae exposed to Cry1Ac when compared to Cry1F. 

### 3.2. Total Distance Flown by Moths

The RFMs used in this study can measure the impact of biotic/abiotic factors on moth dispersal when used in paired comparisons. This laboratory system may not exactly model natural moth environment, but it can provide valuable data on potential dispersal capabilities. The average distances flown by moths depending on strain, sex and diet treatment are shown in Table 2. When larvae were fed the control diet, the average distance flown by moths from the Benzon (3272.6 ± 467.7 m) and 456LS4D (3623.5 ± 546.9 m) strains was in line with previous reports of moth flight recorded with RFMs [44,57], and did not differ between the strains (*p* = 0.7201). This observation suggests that the increased pupal weight in 456LS4D compared to Benzon did not affect the distance flown, which could be unexpected considering that larger pupae typically present improved morphological and metabolic attributes for flight and increased dispersal ability [58,59,60,61,62]. The minimum distance flown was similar (Benzon 196.3 m and 456LS4D 174.3 m), while maximum flown distance was clearly longer in 456LS4D (30,746.2 m) compared to Benzon (17,548.2 m) moths. Both of these maximum distances were substantially longer than reported for field *S. frugiperda* (806 m) in mark–release–recapture experiments in Brazil [63]. These differences could be explained by mark–recapture studies failing to capture long-distance dispersal [64] and the lack of field abiotic/biotic factors that affect moth flight tendency from our laboratory estimations using RFMs. 

There was no effect of sex on average flown distance by Benzon moths from larvae reared on the control diet (*p* = 0.7863). In contrast, the average flown distance in the same treatment was significantly longer (*p* = 0.0141) in female (5064.5 ± 933.4 m) compared to male (1992.8 ± 352.0 m) moths in the 456LS4D strain. This sex effect on flight agrees with reports of insect females typically possessing greater migratory capacity than males in multiple species [51,52], including *S. frugiperda* [53]. On the other hand, lack of flown distance differences based on sex were previously reported using RFMs and *S. exigua* moths from larvae fed the control diet [33].

In the Benzon strain, female larvae surviving generally lethal exposure to Cry1Ac emerged into moths that flew significantly longer distance compared to female larvae reared on the control diet (*p* = 0.0101). This phenomenon was not observed in Benzon males (*p* = 0.0821). Increased flown distance was previously reported for *S. exigua* exposed to sublethal Cry1Ac diet [34], but the response was not separately analyzed for sex. Our results suggest that this response is also observed in survivors of generally lethal exposure to Cry1Ac, and likely represents a common response observed in different insect species to avoid laying eggs on a suboptimal or toxic host environment. For instance, poor host quality induces a developmental switch to flight-capable morphs in aphid species [65,66], and increases flight behavior in *Bemisia tabaci* (Gennadius) (Hemiptera: Aleyrodidae) [67] and wood-boring *Batocera rufomaculata* (De Geer) (Coleoptera: Cerambycidae) beetles [68]. Sublethal exposure to chemical pesticides induced increased flight activity in brown planthopper (*Nilaparvata lugens*) (Ståhl) (Hemiptera: Delphacidae) [69]. However, no differences in flight tendency were observed in *H. zea* emerging from conventional and Bt corn plots, even though weight was significantly reduced in pupae from Bt corn plots [54]. 

In the 456LS4D strain, sublethal exposure of larvae to Cry1Ac did not affect the total average distance flown by females (*p* = 0.6530), but resulted in a reduced average distance flown by male moths (*p* = 0.0174) compared to larvae reared on the control diet. In contrast, sublethal exposure to Cry1F in the same strain did not alter the average total distance flown by either female (*p* = 0.2014) or male (*p* = 0.8058) moths compared to moths from larvae reared on the control diet. Interestingly, data from molecular screening for Cry1F-resistance alleles in *S. frugiperda* support that resistance alleles have limited localization. Resistance to Cry1F corn originally described in Puerto Rico [19] was described in the parental strain of 456LS4D and linked to an allele found at a very high frequency (0.4) in the island [23,24]. However, substantial genotyping efforts by multiple groups have failed to detect the resistance allele outside of Puerto Rico, even at Caribbean and migratory destinations in Florida [23,24,26,30]. While speculative, it is plausible that the lack of increased average flight distance in 456LS4D moths after sublethal exposure of larvae to Cry1F helps explain the lack of spread of this resistance allele through migratory pathways. 

### 3.3. Moth Migratory Versus Resident Flight Tendency 

**Figure 1:**
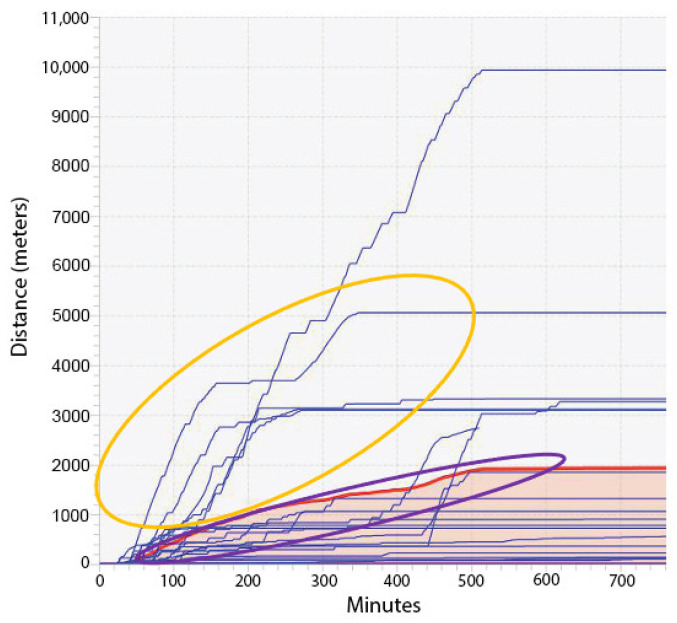
Representative aggregate output from 20 rotation flight mills (RFMs) monitoring distance flown during 12 h by *S. frugiperda* moths. Flight activity for each individual moth is shown as a blue line, while the average flight is shown as a red line with an orange lower area. Moths in this example were from the Benzon strain and were fed control diet as larvae. Orange line circle includes moths that would be classified as “migratory” based on flying >2 km in the first 12 h of testing. Purple line circle includes moths that flew <2 km and would be classified as “resident”.

Insect populations typically present a bimodal flight tendency, previously termed as partial migration, which entails the coexistence of individuals with short (resident) and long-range (migratory) flight tendencies within the same population [70]. There is evidence for both genetic [71] and environmental [72] factors affecting the specific flight behavior (resident versus migratory) displayed by individual insects. This bimodal flight tendency was previously observed in *S. frugiperda* using RFMs, with a strong migratory tendency observed in 58% of field-collected individuals [44]. Adults of both Benzon and 456LS4D strains fed the control diet as larvae also displayed bimodal flight distributions (Figure 1), with moths presenting either short (<2 km) or long (up to 30.7 km)-distance flights within the first 12 h of monitored flight. 

**Figure 2:**
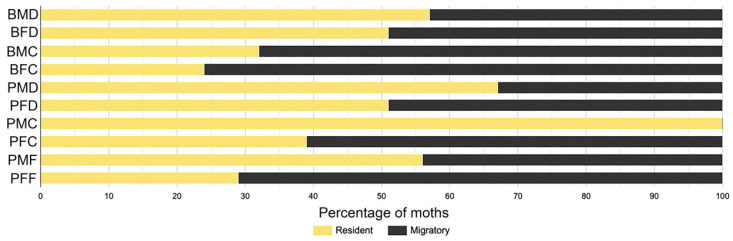
Percentage of moths in the resident (flying < 2 km in 12 h, yellow) and migratory (flying > 2 km in 12 h, black) categories depending on strain, sex and diet treatment during the larval stage. Samples are named on the left using one letter abbreviation for strain (B = Benzon, P = 456LS4D), sex (M = male, F = female), and diet treatment (D = control diet, C = Cry1Ac, F = Cry1F).

Based on the bimodal flight tendency observed, we analyzed flight data by parsing *S. frugiperda* moths from each population into two discrete categories, resident (flying less than 2 km) and migratory (flying more than 2 km) moths (Figure 2). The number of moths in each flight category for each strain when fed the control diet as larvae did not differ between strains (*p* = 0.406), which is in agreement with the lack of strain differences in total flown distance. Again, this observation suggests that moths emerging from the heavier 456LS4D pupae display similar flight tendency compared to moths from lighter Benzon pupae. In both Benzon and 456LS4D strains, most moths from larvae fed the control diet displayed resident flight tendency (53% for Benzon and 59% for 456LS4D). When compared based on sex, female moths from larvae fed the control diet displayed slightly higher tendency than males for migratory flight in both strains (48.6% of females versus 43.3% of males in Benzon; 48.8% versus 32.7% of males in 456LS4D).

When evaluating the data for both sexes in survivors from a generally lethal exposure to Cry1Ac, we detected an overall 7.6-fold increased probability for Benzon moths displaying migratory behavior (*p* = 0.0054, odds ratio = 8.5785) compared to moths from larvae reared on the control diet. However, this shift was only detected in females (*p* = 0.0372), while Benzon male moth flight behavior was not affected when compared to moths from larvae reared on the control diet (*p* = 0.0617). In 456LS4D, sublethal exposure to Cry1Ac did not significantly affect the categorical moth flight tendency distribution compared to moths from larvae reared on the control diet (*p* = 0.9711), independently of sex (females *p* = 0.2404; males *p* = 0.9699). Similarly, sublethal exposure to Cry1F compared to the control diet did not affect the flight behavior of 456LS4D moths, independently of sex (females *p* = 0.0519; males *p* = 0.3290). These results agree with the observations of total distance flown and suggest that only in Benzon female moths surviving as larvae a generally lethal exposure to Cry1Ac results in a shift in behavior and increased tendency for longer flight.

Alteration of moth flight tendency after a generally lethal exposure to Cry proteins could have relevant implications for insect resistance management (IRM) tools for Bt crops. The current high dose/refuge IRM approach for Bt crops assumes recessive resistance and that homozygous-resistant adults emerging from Bt crops mate with abundant susceptible moths maintained in nearby non-Bt refuge areas [73]. The resulting heterozygotes are killed by the high levels of toxins produced in the Bt plants, effectively reducing the frequency of resistance alleles and phenotypes in the field. Transgenic Bt plants may produce levels of insecticidal Bt proteins representing a lethal exposure for susceptible *S. frugiperda* [73], but a sublethal treatment for Cry-resistant insects. An increased tendency for longer flight distance in Cry-susceptible females would violate the non-assortative mating expectation with males emerging from the refuge in the high dose/refuge strategy. In addition, increased tendency for longer flown distance could also contribute to increased risk for spread of resistance alleles. However, an important limitation to consider when attempting to extend our RFM observations to predict effects under field conditions is that our experimental protocol did not expose larvae continuously to the Cry toxin throughout the larval period, in contrast to *S. frugiperda* larvae feeding on Bt corn. In addition, the *S. frugiperda* strains used in this study have been maintained under laboratory conditions for >10 years, which may have selected for traits affecting migratory behavior. This possibility would need to be tested using field-collected insects or strains developed from recent field collections. 

In contrast to the response in susceptible moths, the lack of altered moth flight behavior after exposure to Cry1Ac or Cry1F in 456LS4D suggests that the probability of mating with refuge individuals in this case would not be affected. Moreover, lack of changes in the flight behavior also suggests no increased risk of resistance dispersal through migration. As discussed above, available evidence so far supports that practical resistance to Cry1F corn in *S. frugiperda* emerges locally and not because of immigration of resistant individuals. In addition, it is important to consider that currently available Bt corn hybrids produce multiple insecticidal Bt proteins [74], in contrast to the single toxin exposure used in the present study. It could be possible that exposure of Cry1F-resistant *S. frugiperda* to corn producing multiple Bt toxins could result in the same type of escape response detected in Benzon females surviving Cry1Ac exposure. Nonetheless, studies with *H. zea* collected from Bt corn plants producing multiple insecticidal proteins did not detect differences in flight behavior compared to moths from non-Bt corn plants [54]. 

The increased tendency for longer distance flight observed in female moths from the Benzon strain surviving Cry1Ac mirrors previous observations after sublethal exposure to Cry1Ac in *S. exigua* [33]. Shifts between resident and migratory behavior are often associated with different morphological phenotypes [70,71], such as the increased pupal weight in female Benzon moths exposed to Cry1Ac. These phenotype shifts are often related to altered metabolic or energy storage pathways. For instance, the shift between resident and migrant flight behavior in *Mythimma separata* (Walker) (Lepidoptera: Noctuidae) is regulated by juvenile hormone signaling pathways [75]. Future work identifying the molecular switches controlling the change from resident to migratory flight behavior will help identify ways of generally controlling flight tendency in *S. frugiperda* and other insect pests.

## 4. Conclusions

Female moths surviving a generally lethal exposure to Cry1Ac displayed an increased tendency for longer flight. This behavioral shift was not observed in Cry1F-resistant *S. frugiperda*. Longer flight was associated with an increase in pupal weight, suggesting involvement of changes in metabolism and/or energy storage. The increased tendency for longer flights could affect assumptions of the high dose/refuge strategy, given that Bt crops express lethal levels of Cry proteins. Lack of altered flight behavior in Cry1F-resistant *S. frugiperda* may help explain the lack of resistance allele dispersal from Puerto Rico into continental USA. Further work is needed to understand the impact of increased flight behavior after a generally lethal exposure to Cry proteins on resistance management approaches and the physiological and molecular pathways involved in this behavioral shift.

## Figures and Tables

**Table 1:** Mean pupal weights (in milligrams) depending on strain, sex and diet fed as larvae (Control, Cry1Ac and Cry1F). Toxin concentration in diet treatments was 4.75 µg/cm^2^. Data shown are means and corresponding standard errors. n = number of individuals tested.

Strain	Sex	Control	n	Cry1Ac	n	Cry1F	n
Benzon	Female	169.30 ± 4.33	37	186.26 ± 4.39	29	Not tested	--
	Male	177.60 ± 2.73	31	181.77 ± 3.84	31	Not tested	--
456LS4D	Female	183.35 ± 4.08	43	157.89 ± 4.71	38	191.92 ± 4.59	38
	Male	189.63 ± 3.90	53	156.88 ± 8.35	16	196.90 ± 2.68	25

**Table 2:** Average total distance flown (in meters) after 24 h of tethered flight depending on strain, sex, and larval diet (Control, Cry1Ac and Cry1F). Toxin concentration in diet treatments was 4.75 µg/cm^2^. n = number of tested moths.

Strain	Sex	Control	n	Cry1Ac	n	Cry1F	n
Benzon	Female	3287.7 ± 514.8	37	5702.1 ± 862.6	29	Not tested	--
	Male	3537.3 ± 837.5	30	5424.5 ± 1034.7	31	Not tested	--
456LS4D	Female	3023.5 ± 933.4	43	4596.3 ± 789.1	38	6704.3 ± 993.7	38
	Male	2076.0 ± 351.9	53	3796.8 ± 138.1	16	2093.7 ± 348.5	25

## Data Availability

The raw data presented in this study are available in Supplementary Materials.

## Supplementary Materials

The following supporting information can be downloaded at: https://www.mdpi.com/article/10.3390/insects15010007/s1, Table S1: All raw data.

## References

[1] Montezano D.G., Specht A., Sosa-Gomez D.R., Roque-Specht V.F., Sousa-Silva J.C., Paula-Moraes S.V., Peterson J.A., Hunt T.E. (2018). Host plants of *Spodoptera frugiperda* (Lepidoptera: Noctuidae) in the Americas. Afr. Entomol..

[2] Overton K., Maino J.L., Day R., Umina P.A., Bett B., Carnovale D., Ekesi S., Meagher R., Reynolds O.L. (2021). Global crop impacts, yield losses and action thresholds for fall armyworm (*Spodoptera frugiperda*): A review. Crop Protect..

[3] Goergen G., Kumar P.L., Sankung S.B., Togola A., Tamò M. (2016). First report of outbreaks of the fall armyworm *Spodoptera frugiperda* (JE Smith) (Lepidoptera, Noctuidae), a new alien invasive pest in West and Central Africa. PLoS ONE.

[4] Stokstad E. (2017). New crop pest takes Africa at lightning speed. Science.

[5] Sharanabasappa K.C., Asokan R., Swamy H., Maruthi M., Pavithra H., Kavita Hegde S.N., Prabhu S., Goergen G. (2018). First report of the fall armyworm, *Spodoptera frugiperda* (JE Smith) (Lepidoptera: Noctuidae), an alien invasive pest on maize in India. Pest Manag. Hort. Ecosyst..

[6] Sun X.-X., Hu C.-X., Jia H.-R., Wu Q.-L., Shen X.-J., Zhao S.-Y., Jiang Y.-Y., Wu K.-M. (2021). Case study on the first immigration of fall armyworm, *Spodoptera frugiperda* invading into China. J. Integr. Agric..

[7] Navasero M.V., Navasero M.M., Burgonio G.A.S., Ardez K.P., Ebuenga M.D., Beltran M.J.B., Bato M.B., Gonzales P.G., Magsino G.L., Caoili B.L. (2019). Detection of the fall armyworm, *Spodoptera frugiperda* (JE Smith) (Lepidoptera: Noctuidae) using larval morphological characters, and observations on its current local distribution in the Philippines. Philipp. Entomol..

[8] Wu M.F., Qi G.J., Chen H., Ma J., Liu J., Jiang Y.Y., Lee G.S., Otuka A., Hu G. (2021). Overseas immigration of fall armyworm, *Spodoptera frugiperda* (Lepidoptera: Noctuidae), invading Korea and Japan in 2019. Insect Sci..

[9] Cook D.C., Gardiner P.S., Spafford H. (2021). What will fall armyworm (Lepidoptera: Noctuidae) cost western australian agriculture?. J. Econ. Entomol..

[10] Vives Moreno A., Gastón J. (2020). Five new species for the fauna of Spain and other interesting lepidopterological information for Spain and Sudan (Insecta: Lepidoptera). SHILAP Rev. Lepidopt..

[11] Pehlivan S., Atakan E. (2022). First record of the fall armyworm, *Spodoptera frugiperda* (J.E. Smith, 1797) (Lepidoptera: Noctuidae) in Türkiye. Çukurova J. Agric. Food Sci..

[12] Westbrook J., Fleischer S., Jairam S., Meagher R., Nagoshi R. (2019). Multigenerational migration of fall armyworm, a pest insect. Ecosphere.

[13] Li C., Liao J., Ya Y., Liu J., Li J., Yu G. (2022). Analysis of potential distribution of *Spodoptera frugiperda* in western China. J. Asia-Pacif. Entomol..

[14] Paudel Timilsena B., Niassy S., Kimathi E., Abdel-Rahman E.M., Seidl-Adams I., Wamalwa M., Tonnang H.E.Z., Ekesi S., Hughes D.P., Rajotte E.G. (2022). Potential distribution of fall armyworm in Africa and beyond, considering climate change and irrigation patterns. Sci. Rep..

[15] Ramasamy M., Das B., Ramesh R. (2022). Predicting climate change impacts on potential worldwide distribution of fall armyworm based on CMIP6 projections. J. Pest Sci..

[16] Mota-Sanchez D., Wise J.C. Arthropod Pesticide Resistance Database. www.pesticideresistance.org.

[17] Siebert M.W., Tindall K.R., Leonard B.R., Van Duyn J.W., Babcock J.M. (2008). Evaluation of corn hybrids expressing Cry1F (Herculex I Insect Protection) against fall armyworm (Lepidoptera: Noctuidae) in the southern United States. J. Entomol. Sci..

[18] Siebert M.W., Babock J.M., Nolting S., Santos A.C., Adamczyk J.J., Neese P.A., King J.E., Jenkins J.N., McCarty J., Lorenz G.M. (2008). Efficacy of Cry1F insecticidal protein in maize and cotton for control of fall armyworm (Lepidoptera: Noctuidae). Fla. Entomol..

[19] Storer N.P., Babcock J.M., Schlenz M., Meade T., Thompson G.D., Bing J.W., Huckaba R.M. (2010). Discovery and characterization of field resistance to Bt maize: *Spodoptera frugiperda* (Lepidoptera: Noctuidae) in Puerto Rico. J. Econ. Entomol..

[20] Huang F., Qureshi J.A., Meagher R.L., Reisig D.D., Head G.P., Andow D.A., Ni X., Kerns D., Buntin G.D., Niu Y. (2014). Cry1F resistance in fall armyworm *Spodoptera frugiperda*: Single gene versus pyramided Bt maize. PLoS ONE.

[21] Farias J.R., Andow D.A., Horikoshi R.J., Sorgatto R.J., Fresia P., dos Santos A.C., Omoto C. (2014). Field-evolved resistance to Cry1F maize by *Spodoptera frugiperda* (Lepidoptera: Noctuidae) in Brazil. Crop Protect..

[22] Chandrasena D.I., Signorini A.M., Abratti G., Storer N.P., Olaciregui M.L., Alves A.P., Pilcher C.D. (2018). Characterization of field-evolved resistance to *Bacillus thuringiensis*-derived Cry1F delta-endotoxin in *Spodoptera frugiperda* populations from Argentina. Pest Manag. Sci..

[23] Banerjee R., Hasler J., Meagher R., Nagoshi R., Hietala L., Huang F., Narva K., Jurat-Fuentes J.L. (2017). Mechanism and DNA-based detection of field-evolved resistance to transgenic Bt corn in fall armyworm (*Spodoptera frugiperda*). Sci. Rep..

[24] Flagel L., Lee Y.W., Wanjugi H., Swarup S., Brown A., Wang J., Kraft E., Greenplate J., Simmons J., Adams N. (2018). Mutational disruption of the ABCC2 gene in fall armyworm, *Spodoptera frugiperda*, confers resistance to the Cry1Fa and Cry1A.105 insecticidal proteins. Sci. Rep..

[25] Boaventura D., Ulrich J., Lueke B., Bolzan A., Okuma D., Gutbrod O., Geibel S., Zeng Q., Dourado P.M., Martinelli S. (2020). Molecular characterization of Cry1F resistance in fall armyworm, *Spodoptera frugiperda* from Brazil. Insect Biochem. Mol. Biol..

[26] Banerjee R., De Bortoli C.P., Huang F., Lamour K., Meagher R., Buntin D., Ni X., Reay-Jones F.P.F., Stewart S., Jurat-Fuentes J.L. (2022). Large genomic deletion linked to field-evolved resistance to Cry1F corn in fall armyworm (*Spodoptera frugiperda*) from Florida. Sci. Rep..

[27] Jakka S.R., Knight V.R., Jurat-Fuentes J.L. (2014). Fitness costs associated with field-evolved resistance to Bt maize in *Spodoptera frugiperda* (Lepidoptera: Noctuidae). J. Econ. Entomol..

[28] Vélez A.M., Spencer T.A., Alves A.P., Crespo A.L.B., Siegfried B.D. (2013). Fitness costs of Cry1F resistance in fall armyworm, *Spodoptera frugiperda*. J. Appl. Entomol..

[29] Storer N.P., Kubiszak M.E., Ed King J., Thompson G.D., Santos A.C. (2012). Status of resistance to Bt maize in *Spodoptera frugiperda*: Lessons from Puerto Rico. J. Invertebr. Pathol..

[30] Tandy P., Lamour K., Placidi de Bortoli C., Nagoshi R., Emrich S.J., Jurat-Fuentes J.L. (2023). Screening for resistance alleles to Cry1 proteins through targeted sequencing in the native and invasive range of *Spodoptera frugiperda* (Lepidoptera: Noctuidae). J. Econ. Entomol..

[31] Benard M.F., McCauley S.J. (2008). Integrating across life-history stages: Consequences of natal habitat effects on dispersal. Am. Nat..

[32] Mazzi D., Dorn S. (2012). Movement of insect pests in agricultural landscapes. Ann. Appl. Biol..

[33] Jiang X.F., Chen J., Zhang L., Sappington T.W., Luo L.Z. (2013). Increased long-flight activity triggered in beet armyworm by larval feeding on diet containing Cry1Ac protoxin. PLoS ONE.

[34] Jakka S.R.K., Gong L., Hasler J., Banerjee R., Sheets J.J., Narva K., Blanco C.A., Jurat-Fuentes J.L. (2016). Field-evolved Mode 1 resistance of the fall armyworm to transgenic Cry1Fa-expressing corn associated with reduced Cry1Fa toxin binding and midgut alkaline phosphatase expression. Appl. Environ. Microbiol..

[35] Abdelgaffar H., Tague E.D., Castro Gonzalez H.F., Campagna S.R., Jurat-Fuentes J.L. (2019). Midgut metabolomic profiling of fall armyworm (*Spodoptera frugiperda*) with field-evolved resistance to Cry1F corn. Insect Biochem. Mol. Biol..

[36] Zhao C., Jurat-Fuentes J.L., Abdelgaffar H.M., Pan H., Song F., Zhang J. (2015). Identification of a new cry1I-type gene as a candidate for gene pyramiding in corn to control *Ostrinia* species larvae. Appl. Environ. Microbiol..

[37] Gouffon C., Van Vliet A., Van Rie J., Jansens S., Jurat-Fuentes J.L. (2011). Binding sites for *Bacillus thuringiensis* Cry2Ae toxin on heliothine brush border membrane vesicles are not shared with Cry1A, Cry1F, or Vip3A toxin. Appl. Environ. Microbiol..

[38] Abdelgaffar H., Perera O.P., Jurat-Fuentes J.L. (2021). ABC transporter mutations in Cry1F-resistant fall armyworm (*Spodoptera frugiperda*) do not result in altered susceptibility to selected small molecule pesticides. Pest Manag. Sci..

[39] Jakka S.R., Knight V.R., Jurat-Fuentes J.L. (2014). *Spodoptera frugiperda* (J. E. Smith) with field-evolved resistance to Bt maize are susceptible to Bt pesticides. J. Invertebr. Pathol..

[40] Deshmukh S., Prasanna B., Kalleshwaraswamy C., Jagdish J., Choudhary B. (2021). Fall armyworm *Spodoptera frugiperda* (J. E. Smith). Omkar (Ed) Polyphagous Pest of Corn.

[41] Jones V.P., Naranjo S.E., Smith T.J. Insect Ecology and Behavior Laboratory: Flight Mill Studies. https://tfrec.cahnrs.wsu.edu/vpjones/flight-mill-studies/.

[42] Beerwinkle K.R., Lopez J.D., Cheng D., Lingren P.D., Meola R.W. (1995). Flight potential of feral *Helicoverpa zea* (Lepidoptera: Noctuidae) males measured with a 32-channel, computer-monitored, flight-mill system. Environ. Entomol..

[43] Naranjo S.E. (2019). Assessing insect flight behavior in the laboratory: A primer on flight mill methodology and what can be learned. Ann. Entomol. Soc. Am..

[44] Chen H., Wang Y., Huang L., Xu C.F., Li J.H., Wang F.Y., Cheng W., Gao B.Y., Chapman J.W., Hu G. (2022). Flight capability and the low temperature threshold of a Chinese field population of the fall armyworm *Spodoptera frugiperda*. Insects.

[45] Ge S.-S., He L.-M., He W., Yan R., Wyckhuys K.A.G., Wu K.-M. (2021). Laboratory-based flight performance of the fall armyworm, *Spodoptera frugiperda*. J. Integr. Agric..

[46] Hietala L., Hietala N., Assirati G., Ferreira dos Santos R., Jurat-Fuentes J.L. (2023). Novel software and hardware package for recording and visualizing rotational insect flight mill data. J. Biol. Meth..

[47] Moar W., Khajuria C., Pleau M., Ilagan O., Chen M., Jiang C., Price P., McNulty B., Clark T., Head G. (2017). Cry3Bb1-resistant Western corn rootworm, *Diabrotica virgifera virgifera* (LeConte) does not exhibit cross-resistance to DvSnf7 dsRNA. PLoS ONE.

[48] Mishra S., Dee J., Moar W., Dufner-Beattie J., Baum J., Dias N.P., Alyokhin A., Buzza A., Rondon S.I., Clough M. (2021). Selection for high levels of resistance to double-stranded RNA (dsRNA) in Colorado potato beetle (*Leptinotarsa decemlineata* Say) using non-transgenic foliar delivery. Sci. Rep..

[49] Salama H.S., Foda M.S., El-Sharaby A., Matter M., Khalafallah M. (1981). Development of some lepidopterous cotton pests as affected by exposure to sublethal levels of endotoxins of *Bacillus thuringiensis* for different periods. J. Invertebr. Pathol..

[50] Pedersen A., Dedes J., Gauthier D., Van Frankenhuyzen K. (1997). Sublethal effects of *Bacillus thuringiensis* on the spruce budworm, *Choristoneura fumiferana*. Entomol. Exp. Appl..

[51] Stapel J.O., Waters D.J., Ruberson J.R., Lewis W.J. (1998). Development and behavior of *Spodoptera exigua* (Lepidoptera: Noctuidae) larvae in choice tests with food substrates containing toxins of *Bacillus thuringiensis*. Biol. Control.

[52] Sayyed A.H., Cerda H., Wright D.J. (2003). Could Bt transgenic crops have nutritionally favourable effects on resistant insects?. Ecol. Lett..

[53] Tabashnik B.E., Carrière Y. (2004). Bt transgenic crops do not have favorable effects on resistant insects. J. Insect Sci..

[54] Pezzini D.T., Reisig D.D., Buntin G.D., Del Pozo-Valdivia A.I., Gould F., Paula-Moraes S.V., Reay-Jones F.P. (2023). Impact of seed blend and structured maize refuge on *Helicoverpa zea* (Lepidoptera: Noctuidae) potential phenological resistance development parameters in pupae and adults. Pest Manag. Sci..

[55] Dively G.P., Venugopal P.D., Finkenbinder C. (2016). Field-evolved resistance in corn earworm to Cry proteins expressed by transgenic sweet corn. PLoS ONE.

[56] Carrière Y., Tabashnik B.E. (2023). Fitness costs and incomplete resistance associated with delayed evolution of practical resistance to Bt crops. Insects.

[57] Jones H.B.C., Lim K.S., Bell J.R., Hill J.K., Chapman J.W. (2016). Quantifying interspecific variation in dispersal ability of noctuid moths using an advanced tethered flight technique. Ecol. Evol..

[58] Su S., Wang X., Jian C., Ignatus A.D., Zhang X., Peng X., Chen M. (2021). Life-history traits and flight capacity of *Grapholita molesta* (Lepidoptera: Tortricidae) using artificial diets with varying sugar content. J. Econ. Entomol..

[59] Su S., Zhang X., Jian C., Huang B., Peng X., Vreysen M.J.B., Chen M. (2022). Effects of adult feeding treatments on longevity, fecundity, flight ability, and energy metabolism enzymes of *Grapholita molesta* moths. Insects.

[60] Jahant-Miller C., Miller R., Parry D. (2022). Size-dependent flight capacity and propensity in a range-expanding invasive insect. Insect Sci..

[61] Elliott C.G., Evenden M.L. (2009). Factors influencing flight potential of *Choristoneura conflictana*. Physiol. Entomol..

[62] Huang J., Gao L., Cobb T., Li G., Tian C., Duan A., Feng H. (2023). The effect of larval diet on the flight capability of the adult moth *Athetis lepigone* (Möschler) (Lepidoptera: Noctuidae). Fla. Entomol..

[63] Vilarinho E.C., Fernandes O.A., Hunt T.E., Caixeta D.F. (2011). Movement of *Spodoptera frugiperda* adults (Lepidoptera: Noctuidae) in maize in Brazil. Fla. Entomol..

[64] Robinet C., David G., Jactel H. (2019). Modeling the distances traveled by flying insects based on the combination of flight mill and mark-release-recapture experiments. Ecol. Model..

[65] Forrest J.M.S. (1970). The effect of maternal and larval experience on morph determination in *Dysaphis devecta*. J. Insect Physiol..

[66] Mackay P.A., Lamb R.J. (1996). Disperal of five aphids (Homoptera: Aphididae) in relation to their impact on *Hordeum vulgare*. Environ. Entomol..

[67] Blackmer J.L., Byrne D.N. (1993). Flight behaviour of *Bemisia tabaci* in a vertical flight chamber: Effect of time of day, sex, age and host quality. Physiol. Entomol..

[68] Brown S., Soroker V., Ribak G. (2017). Effect of larval growth conditions on adult body mass and long-distance flight endurance in a wood-boring beetle: Do smaller beetles fly better?. J. Insect Physiol..

[69] Zhao K.F., Shi Z.P., Wu J.C. (2011). Insecticide-induced enhancement of flight capacity of the brown planthopper *Nilaparvata lugens* Stål (Hemiptera: Delphacidae). Crop Protect..

[70] Menz M.H.M., Reynolds D.R., Gao B., Hu G., Chapman J.W., Wotton K.R. (2019). Mechanisms and consequences of partial migration in insects. Front. Ecol. Evo..

[71] Dällenbach L.J., Glauser A., Lim K.S., Chapman J.W., Menz M.H.M. (2018). Higher flight activity in the offspring of migrants compared to residents in a migratory insect. Proc. Royal Soc. B Biol. Sci..

[72] Becciu P., Menz M.H.M., Aurbach A., Cabrera-Cruz S.A., Wainwright C.E., Scacco M., Ciach M., Pettersson L.B., Maggini I., Arroyo G.M. (2019). Environmental effects on flying migrants revealed by radar. Ecography.

[73] Reisig D.D., DiFonzo C., Dively G., Farhan Y., Gore J., Smith J. (2022). Best management practices to delay the evolution of Bt resistance in lepidopteran pests without high susceptibility to Bt toxins in North America. J. Econ. Entomol..

[74] Gassmann A.J., Reisig D.D. (2023). Management of insect pests with Bt crops in the United States. Annu. Rev. Entomol..

[75] Zhang L., Cheng L., Chapman J.W., Sappington T.W., Liu J., Cheng Y., Jiang X. (2020). Juvenile hormone regulates the shift from migrants to residents in adult oriental armyworm, *Mythimna separata*. Sci. Rep..

